# Effects of Organic Polymer Compound Material on K^+^ and Na^+^ Distribution and Physiological Characteristics of Cotton Under Saline and Alkaline Stresses

**DOI:** 10.3389/fpls.2021.636536

**Published:** 2021-05-28

**Authors:** Xiaoli Wang, Mengjie An, Kaiyong Wang, Hua Fan, Jiaohua Shi, Kuan Chen

**Affiliations:** Agricultural College, Shihezi University, Shihezi, China

**Keywords:** composite material, saline and alkaline stresses, girdling, K^+^/Na^+^ ratio, physiological characteristics

## Abstract

Soil salinization and alkalization greatly restrict crop growth and yield. In this study, NaCl (8 g kg^−1^) and Na_2_CO_3_ (8 g kg^−1^) were used to create saline stress and alkaline stress on cotton in pot cultivation in the field, and organic polymer compound material (OPCM) and stem girdling were applied before cotton sowing and at flowering and boll-forming stage, respectively, aiming to determine the effects of OPCM on K^+^ and Na^+^ absorption and transport and physiological characteristics of cotton leaf and root. The results showed that after applying the OPCM, the Na^+^ content in leaf of cotton under saline stress and alkaline stress were decreased by 7.72 and 6.49%, respectively, the K^+^/Na^+^ ratio in leaf were increased by 5.65 and 19.10%, respectively, the Na^+^ content in root were decreased by 9.57 and 0.53%, respectively, the K^+^/Na^+^ ratio in root were increased by 65.77 and 55.84%, respectively, and the transport coefficients of K^+^ and Na^+^ from leaf to root were increased by 39.59 and 21.38%, respectively. The activities of superoxide dismutase (SOD), catalase (CAT), and peroxidase (POD), and the relative electrical conductivity (REC) in cotton leaf were significantly increased, while the content of malondialdehyde (MDA) was decreased; but the changes in those in root were not significant. The boll weights were increased by 11.40 and 13.37%, respectively, compared with those for the control. After stem girdling, the application of OPCM still promoted the ion transport of cotton organs; moreover, the CAT activity in root was increased by 25.09% under saline stress, and the SOD activity in leaf and CAT in root were increased by 42.22 and 6.91%, respectively under alkaline stress. Therefore, OPCM can significantly change the transport of K^+^ and Na^+^ to maintain the K^+^ and Na^+^ homeostasis in leaf and root, and regulate physiological and biochemical indicators to alleviate the stress-induced damage. Besides, the regulation effect of OPCM on saline stress was better than that on alkaline stress.

## Introduction

Soil salinization causes abiotic stress to crops and greatly limits crop growth. It has become one of the global environmental problems for agriculture production (Haider et al., 2019). Neutral salt (NaCl and Na_2_SO_4_) and alkaline salt (Na_2_CO_3_ and NaHCO_3_) are the main components in salinized and alkalized soils, respectively (Alvarez-Acosta et al., 2019). According to the report, the area of salinized and alkalized soils in the world are about 4.34 and 3.97 million hectares, respectively (Xu et al., 2020). The negative effect of alkaline stress on ecological environment is greater than that of saline stress (Cheng et al., 2018). Unreasonable irrigation of farmland soil also leads to the soil salinization, which could seriously affect the growth of crops (Hussain et al., 2019). Therefore, the comprehensive management of salinized and alkalized soils and the improvement of saline-alkaline tolerance of crops have become important issues in environmental management and agricultural development.

Cotton is one of the important economic crops in the world. However, saline and alkaline stresses have caused low emergence rate and low boll set rate in cotton cultivation in many regions (Wei et al., 2017; Chen et al., 2021). Previous study has found that applying exogenous substances is a simple and effective way to improve the saline and alkaline tolerance of crops (Ding et al., 2019). Exogenous application of salicylic acid (SA) and nitric oxide (NO) could effectively promote the growth of crops (Ahanger et al., 2019), and exogenous application of lignin could increase cotton yield by 15.6–21.1% in salinized soil (Liu et al., 2019). In addition, girdling could also improve the saline and alkaline tolerance of cotton. Girdling is a technique used for interrupting the transport of assimilates through the phloem without influencing water flow in the xylem in physiological studies. It makes assimilates accumulated above the cut (Poirier-Pocovi et al., 2018). Vemmos et al. (2012) have shown that girdling has significant effects in controlling the vegetative growth of fruit trees and promoting the flowering and fruiting. The content of mineral elements in plant organs could also be reduced by girdling. That is, the transport and accumulation of mineral elements in plant organs could be affected by girdling. However, the current researches mostly focus on the effect of salt stress on ion accumulation in plants in indoor culture experiments (Ahanger et al., 2018), while the research on saline and alkaline tolerance of plants in field environment is less.

This study used cotton in the flowering and boll-forming stage as the study material, combined with stem girdling, to explore the effects of organic polymer compound material (OPCM) on K^+^ and Na^+^ transport and accumulation as well as oxidative damage in cotton organs under saline and alkaline stresses. We hypothesized: (1) the K^+^ and Na^+^ transport and physiological regulation in cotton organs might be different under saline and alkaline stresses; (2) OPCM might regulate the transport of K^+^ and Na^+^ in cotton organs; and (3) OPCM might maintain the ion homeostasis through regulating catalase (CAT) activity in cotton leaf and root to reduce saline and alkaline stresses on cotton. This study helps us understand how OPCM regulates ion transport and physiological characteristics of crops under saline and alkaline stresses, and provides a good method for reducing the saline and alkaline stresses on cotton cultivated in arid areas.

## Materials and Methods

### Test Site

The experiment was conducted in Shihezi Grape Research Institute (44°20' N, 86°03' E) in 2018 and 2019. The climate is temperate continental. The test soil is gray desert soil, with soil pH of 7.72, soil cation exchange capacity of 17.32 cmol kg^−1^, soil organic matter of 12.5 g kg^−1^, alkali-hydrolyzed nitrogen of 54 mg kg^−1^, available phosphorus of 11.7 mg kg^−1^, and available potassium of 218 mg kg^−1^. The cotton variety is Xinluzao 62.

### Materials and Experimental Design

On April 25, 2018, salinization and alkalization of soils were carried out in the way of applying NaCl and Na_2_CO_3_, respectively, making the soil salt content reach 8 g kg^−1^. The pH value of salinized soil was 8.24, and soil electric conductivity (1:5) was 4.24 ds m^−1^; the pH value of alkalized soil was 9.78, and soil electric conductivity (1:5) was 2.78 ds m^−1^. On April 20, 2019, salinization and alkalization of soils were carried out in the same way, making the soil salt content reach 8 g kg^−1^. The pH value of salinized soil was 8.11, and soil electric conductivity (1:5) was 4.83 ds m^−1^; the pH value of alkalized soil was 9.62, and soil electric conductivity (1:5) was 2.57 ds m^−1^. There were eight treatments in total, S: salinized soil; A: alkalized soil (Table 1). OPCM used in this study was a mixture of calcium lignosulfonate, manganese sulfate, zinc sulfate, ferric sulfate, boric acid, anionic polyacrylamide, and polyvinyl alcohol (mass ratio: 4: 4: 4: 2: 1: 0.5: 0.5).

**Table 1:** Test design.

Group	Phloem girdling	Compound material application
S	No girdling	No added
A	No girdling	No added
S1	No girdling	Organic polymer compound material
A1	No girdling	Organic polymer compound material
S-J	Stem girdling	No added
A-J	Stem girdling	No added
S1-J	Stem girdling	Organic polymer compound material
A1-J	Stem girdling	Organic polymer compound material

Salinized soil and alkalized soil were separately put into barrels (50 cm in diameter and 60 cm in height), and barrels were buried in the field on April 2018 and 2019. Urea of 360 kg hm^−2^ and compound fertilizer (N: P_2_O_5_: K_2_O = 20: 9: 9) of 795 kg hm^−2^ were applied on April, 2018 and 2019. Cotton was sown in barrels on May 4. OPCM (300 kg hm^−2^) was dissolved in water and irrigated before seedling emergence on June 25. After emergence, six plants were retained per barrel. During the whole growth period, the total irrigation volume was 4,500 m^3^ hm^−2^, and the irrigated cycle was 10 days. Nine times of irrigation totally were conducted. No fertilizers and OPCM were applied in the later. On July 25 (in the flowering and boll-forming stage), girdling (width: 1 cm) on cotton main stem was performed. Five plant samples were collected from each treatment for the determination of physiological and biochemical indicators on August 10, 2018, and three cotton plants were randomly collected from different plots to determine the ion content. On August 5, 2019, three cotton plants were collected from the barrels to determine the growth indexes.

### Measurement of Growth Index

Samples were collected in the flowering and boll-forming stage in 2018 and 2019, and three plants from each treatment were collected and taken back to the lab. Plants were washed, and root, stem (including leaf sheath), and leaf were isolated. After that, organs were dried in an oven at 105°C for 30 min, and then dried at 75°C to the constant weight. Meanwhile, the boll weight of six cotton plants from each treatment was determined.

### Measurement of Malondialdehyde and Relative Electrical Conductivity

The content of thiobarbituric acid reactants (TBARS) was measured according to the method of Heath and Packer (1968) to calculate the content of malondialdehyde (MDA; umol g^−1^; extinction coefficient: 155 mm cm^−1^). Fresh sample (0.5 g) of each organ was ground to homogenate and extracted in 10 ml of 0.6% TBA (made with 10% trichloroacetic acid). Then, the extract was heated at 100°C for 30 min, cooled on ice, and centrifuged at 5,000 r min^−1^ for 10 min. After that, the absorbance was measured at 532 nm. Non-specific turbidity was corrected by subtracting the absorbance value at 600 nm. Relative electrical conductivity was measured according to the method of Lutts et al. (1996). Fresh sample (0.1 g) of each organ was put in a closed test tube containing 10 ml of deionized water to measure conductivity (R1) using a conductivity meter (DDS-11A, Shanghai Leici Instrument Co., Ltd.), after shaking for 24 h at room temperature on a rotary shaker (QL200H, Shanghai Qiqiang Instrument Co., Ltd.). Then, the sample was boiled in a water bath for 30 min and the conductivity was determined again (R2). The calculation formula was as follows:

$$\mathrm{REC}\;\left(\%\right)=\frac{R1}{R2}\times 100\%$$

### Measurement of Enzymes of Antioxidant System

Preparation of enzyme extract: Potassium phosphate buffer (5 ml, 50 mM, pH: 7.8) was added to 0.5 g sample of each organ and ground to homogenate after an ice bath (An et al., 2019). The homogenate was centrifuged at 10,000 r min^−1^ for 20 min at 4°C, and the enzyme activity was measured spectrophotometrically using the supernatant. Superoxide dismutase (SOD) activity was measured with the method of photochemical reduction in NBT proposed by Paoletti et al. (1986). The reaction mixture (3 ml) consisted of 0.05 M potassium phosphate buffer (pH: 7.8), 130 mM methionine, 750 μM NBT, 20 μM riboflavin, 100 μM EDTA-Na_2_, and 200 μl enzyme extract. SOD activity was measured at 560 nm (one unit of SOD activity was represented by the amount of enzyme required to cause 50% inhibition of NBT photochemical reduction). Peroxidase (POD) activity was measured at 470 nm with the method of Zhou and Leul (1999). The reaction mixture consisted of 0.1 M potassium phosphate buffer (pH: 6.0), guaiacol, H_2_O_2_, and 100 μl enzyme extract. CAT activity was measured in a 2.5 ml reaction solution containing 0.1 M potassium phosphate buffer (pH: 7.0) and 0.1 M H_2_O_2_ at 240 nm within 1 min (Cakmak and Marschner, 1992).

### Measurement of Soluble Sugar and Soluble Protein Content

The soluble sugar content was measured according to the method of Hizukuri et al. (1989). Fresh sample (1 g) of each organ was ground with 10 ml of distilled water to homogenate, and then the homogenate was boiled in a water bath for 10 min. After cooling, the supernatant (0.2 ml) was pipetted and 5 ml of anthrone-sulfuric acid reagent was added, following by the boiling in a water bath for 10 min. After cooling to room temperature, a standard curve was prepared with glucose solution, and the absorbance was measured at 620 nm.

The soluble protein content was measured according to the method of Read and Northcote (1981). Fresh sample (0.5 g) of each organ was ground with 5 ml of distilled water to homogenate, and the homogenate was centrifuged at 3,000 r min^−1^ for 10 min. Then, 0.3 ml of the extract was pipetted and 5 ml of Coomassie Brilliant Blue was added. The absorbance was measured at 595 nm after 2 min.

### Measurement of K^+^ and Na^+^ Content in Cotton Organs

Dried organ samples were pulverized. After that, 0.1 g pulverized sample was weighed, and 5 ml 98% H_2_SO_4_ and 300 g·l^−1^ H_2_O_2_ were added. The solution was heated until the liquid become transparent, and then distilled water was added to make the volume to 100 ml. After that, 5 ml of test solution was pipetted, and distilled water was added to make the volume to 50 ml. Finally, the solution was measured with flame photometer (FP6410 flame photometer, Shanghai Yidian Scientific Instrument Co., Ltd., China; Bao, 2000).

### Selective Transport (ST_K-Na_) of K^+^ and Na^+^ in Cotton Organs

Selective transport of K^+^ and Na^+^ (ST_K-Na_) in different cotton organs was measured according to the method proposed by Brown (1998):

$$ST\left(a/b\right)=\frac{\;K^{+}/{\mathrm{Na}}^{+}\;\mathrm{of organ}\;a}{\;K^{+}/{\mathrm{Na}}^{+}\;\mathrm{of organ}\;b}$$

The higher the *ST* value, the higher the selective transport of K^+^ or Na^+^ from organ b to organ a. In this study, the selective transports of K^+^ and Na^+^ from leaf to stem and from stem to root were determined.

### Data Statistics and Analysis

Excel 2010 and Origin 8.5 software were used to process data and draw charts, respectively. One-way ANOVA and Duncan multiple-range tests in SPSS 23.0 software were used to test the significance of differences at *p* < 0.05. Redundancy analysis (RDA) was conducted using RStudio software.

## Results

### Effects of OPCM on the Growth Indexes of Cotton Under Saline and Alkaline Stresses

The plant height, root length, and total dry matter of cotton for the S1 and S treatments were higher than those for the A1 and A treatments; and the plant height, root length, and total dry matter for the S1 and A1 treatments were higher than those for the S and A treatments (Table 2). There was no significant difference in the plant height and root length between S1 and A1 treatments and A treatment. The boll weight for the S1 treatment was insignificantly increased by 11.40% compared with that for the S treatment, and the boll weight for the A1 treatments was insignificantly increased by 13.37% compared with that for the A treatment.

**Table 2:** Effects of compound material on the growth indexes of cotton under saline and alkaline stresses.

Year	Treat	Plant height (cm)	Root length (cm)	Total dry matter (g)	Boll weight (g barrel^−1^)
2018	S	74.25 ± 1.77ab	23.00 ± 2.83ab	54.04 ± 1.01bc	216.70 ± 10.49b
	S1	77.00 ± 1.41a	26.50 ± 2.12a	60.60 ± 3.97a	250.14 ± 7.36a
	A	71.50 ± 2.12b	19.00 ± 2.83b	50.41 ± 1.12c	218.22 ± 9.06b
	A1	74.75 ± 3.89ab	23.75 ± 2.47ab	57.86 ± 0.53ab	246.29 ± 12.08a
2019	S	59.40 ± 1.42a	19.09 ± 2.35a	45.93 ± 0.86ab	175.52 ± 8.50b
	S1	63.14 ± 1.16a	21.47 ± 1.72a	49.69 ± 2.78a	210.12 ± 6.18a
	A	57.92 ± 1.72a	16.15 ± 2.41ab	42.34 ± 0.95b	185.49 ± 7.70b
	A1	62.79 ± 2.72a	19.71 ± 2.05a	48.60 ± 0.45a	201.96 ± 9.43ab

S, S1 indicate no compound material was applied in salinized soil, organic polymer was applied in salinized soil, respectively; A, A1 indicate no compound material was applied in alkalized soil, organic polymer was applied in alkalized soil, respectively. Values are means ± SD of three independent replications (*n* = 3). Different lower-case letters indicate that there were significant differences among treatments at the level of 0.05.

### Effects of OPCM on Physiological and Biochemical Characteristics of Cotton Under Saline and Alkaline Stresses, Coupled With Stem Girdling

#### MDA and Relative Electrical Conductivity in Cotton Organs

**Figure 1:**
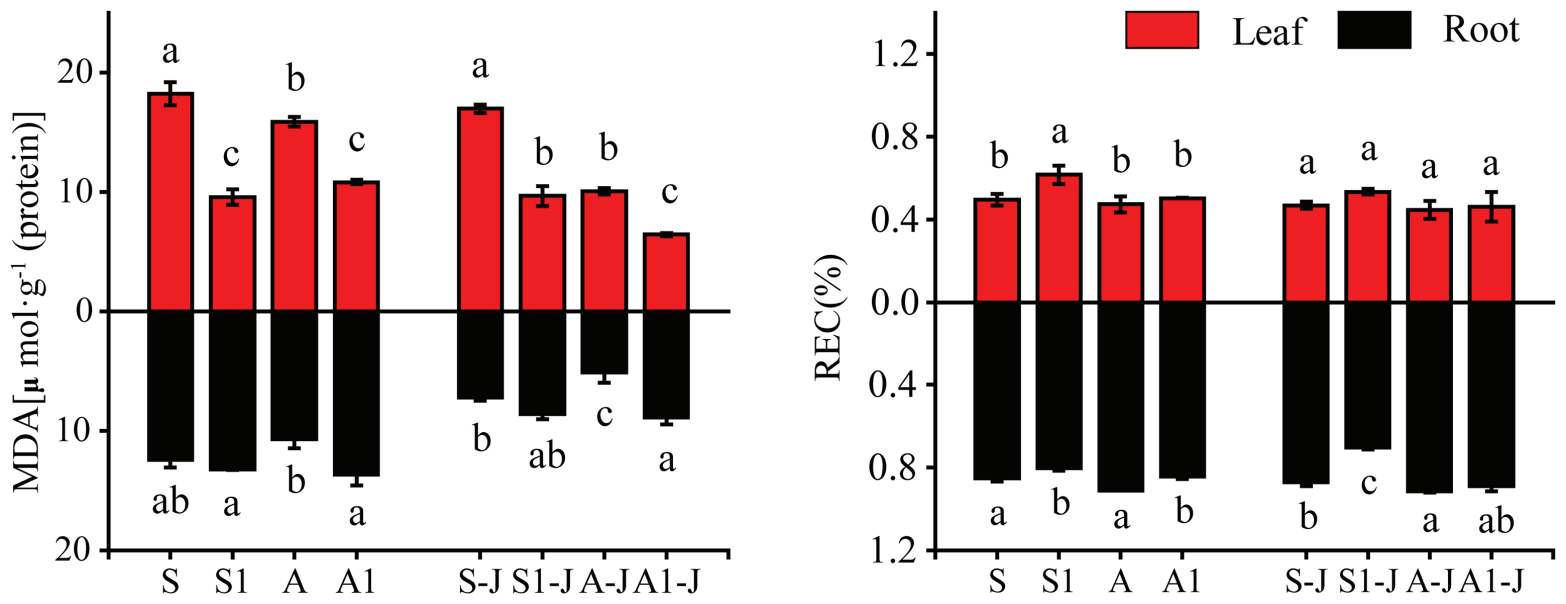
Effects of compound material on malondialdehyde (MDA) and relative electrical conductivity (REC) of cotton leaf and root under saline and alkaline stresses. S, S1 indicate no compound material was applied in salinized soil, organic polymer was applied in salinized soil, respectively; A, A1 indicate no compound material was applied in alkalized soil, organic polymer was applied in alkalized soil, respectively. Bars represent SD of the mean (*n* = 3). Different lower-case letters indicate that there were significant differences among treatments at the level of 0.05.

For non-girdling cottons, the MDA content in root for the A treatment was significantly decreased by 14.08%, and the MDA content in leaf for the S1 treatment was significantly decreased by 47.50%, compared with that for the S treatment. No significant differences were found in the MDA content in leaf between the S treatment and A treatment and the MDA content in root between the S1 treatment and the S treatment. The MDA content in leaf for the A1 treatment was significantly decreased by 31.85%, while the MDA content in root was significantly increased by 27.68%, compared with those for the A treatment. For stem-girdling cottons, the MDA content in leaf for the S1-J treatment and the A1-J treatment were significantly decreased by 43.00 and 35.96%, respectively, while the MDA content in root were significantly increased by 19.61 and 73.73%, respectively, compared with those for the A-J treatment (Figure 1).

For non-girdling cottons, there were no significant differences in the relative conductivity in leaf and root between the A treatment and the S treatment. The relative conductivity in leaf for the S1 treatment was significantly increased by 24.02% compared with that for the S treatment, while no significant difference was found between the A1 treatment and the A treatment. The relative conductivity in root for the S1 treatment was significantly decreased by 6.00% compared with that for the S treatment, and the relative conductivity in root for the A1 treatment was significantly decreased by 7.45% compared with that for the A treatment. For stem-girdling cottons, there were no significant differences in the relative conductivity in leaf and root between the A1-J treatment and the A-J treatment. However, the relative conductivity in root for the S1-J treatment was significantly decreased by 19.00% compared with that for the S-J treatment, and no significant difference was found in the relative conductivity in leaf (Figure 1).

#### Antioxidant Enzyme Activities in Cotton Organs

For non-girdling cottons, the SOD activity in leaf for the A treatment was significantly increased by 21.29% compared with that for the S treatment, and no significant difference was found in the SOD activity in root. The SOD activity in leaf for the S1 treatment was significantly increased by 18.93% compared with that for the S treatment, and no significant difference was found in the SOD activity in root. There was also no significant difference in the SOD activity in leaf and root between the A1 treatment and the A treatment. For stem-girdling cottons, the SOD activity in leaf for the A1-J treatment was significantly increased by 42.22% compared with that for the A-J treatment, and no significant difference was found in the SOD activity in root. There was also no significant difference in the SOD activity in leaf and root between the S1-J treatment and the S-J treatment (Figure 3).

**Figure 2:**
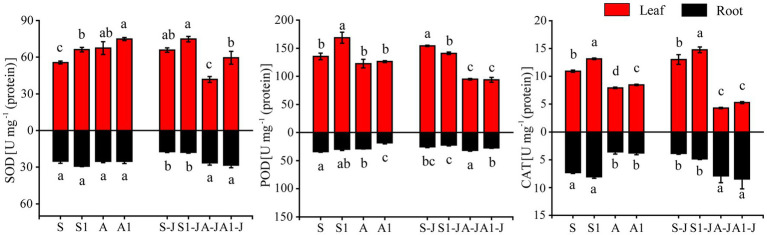
Effects of compound material on Antioxidant enzyme activities of cotton leaf and root under saline and alkaline stresses. S, S1 indicate no compound material was applied in salinized soil, organic polymer was applied in salinized soil, respectively; A, A1 indicate no compound material was applied in alkalized soil, organic polymer was applied in alkalized soil, respectively. Bars represent SD of the mean (*n* = 3). Different lower-case letters indicate that there were significant differences among treatments at the level of 0.05.

**Figure 3:**
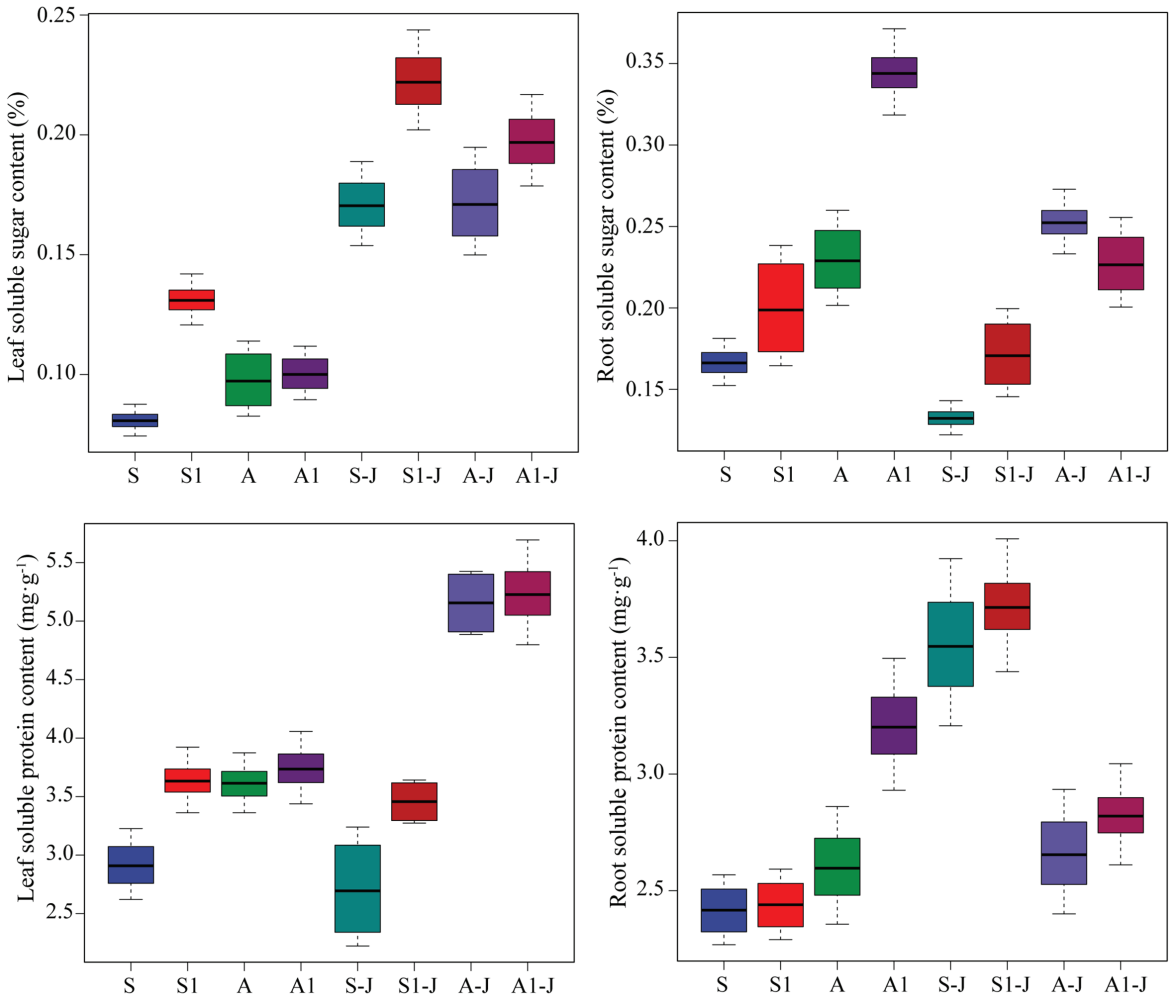
Effects of compound material on osmotic substances in leaf and root of cotton under saline and alkaline stresses. S, S1 indicate no compound material was applied in salinized soil, organic polymer was applied in salinized soil, respectively; A, A1 indicate no compound material was applied in alkalized soil, organic polymer was applied in alkalized soil, respectively. Bars represent SD of the mean (*n* = 3). Different lower-case letters indicate that there were significant differences among treatments at the level of 0.05.

For non-girdling cottons, the POD activity in root for the A treatment was significantly decreased by 15.41% compared with that for the S treatment, and no significant difference was found in the POD activity in leaf. The POD activity in leaf for the S1 treatment was significantly increased by 24.70% compared with those for the S treatment, and the POD activity in root for the A1 treatment was significantly decreased by 37.10% compared with those for the A treatment. No significant differences were found in the POD activity in leaf between the A1 and the A treatment and in the POD activity in root between the S1 and S treatment. For stem-girdling cottons, the POD activity in leaf for the S1-J treatment was significantly decreased by 8.60% compared with that for the S treatment, and no significant difference was found in the POD activity in root. The POD activity in root for the A1-J treatment was significantly decreased by 13.34% compared with that for the A-J treatment, and no significant difference was found in the POD activity in leaf (Figure 3).

For non-girdling cottons, the CAT activity in root for the A treatment was significantly decreased by 51.15% compared with that for the S treatment, and no significant difference was found in the CAT activity in leaf. There were also no significant differences in the CAT activity in leaf and root between the S1 treatment and the S treatment and between the A1 treatment and the A treatment. For stem-girdling cottons, the CAT activity in root for the S1-J treatment was increased by 25.09% compared with that for the S-J treatment, and the CAT activity in root for the A1-J treatment were increased by 6.91% compared with that for the A-J treatment, and no significant difference was found in the CAT activity in leaf between the S1-J treatment and the A1-J treatment (Figure 3).

#### Osmotic Substances in Cotton Organs

**Figure 4:**
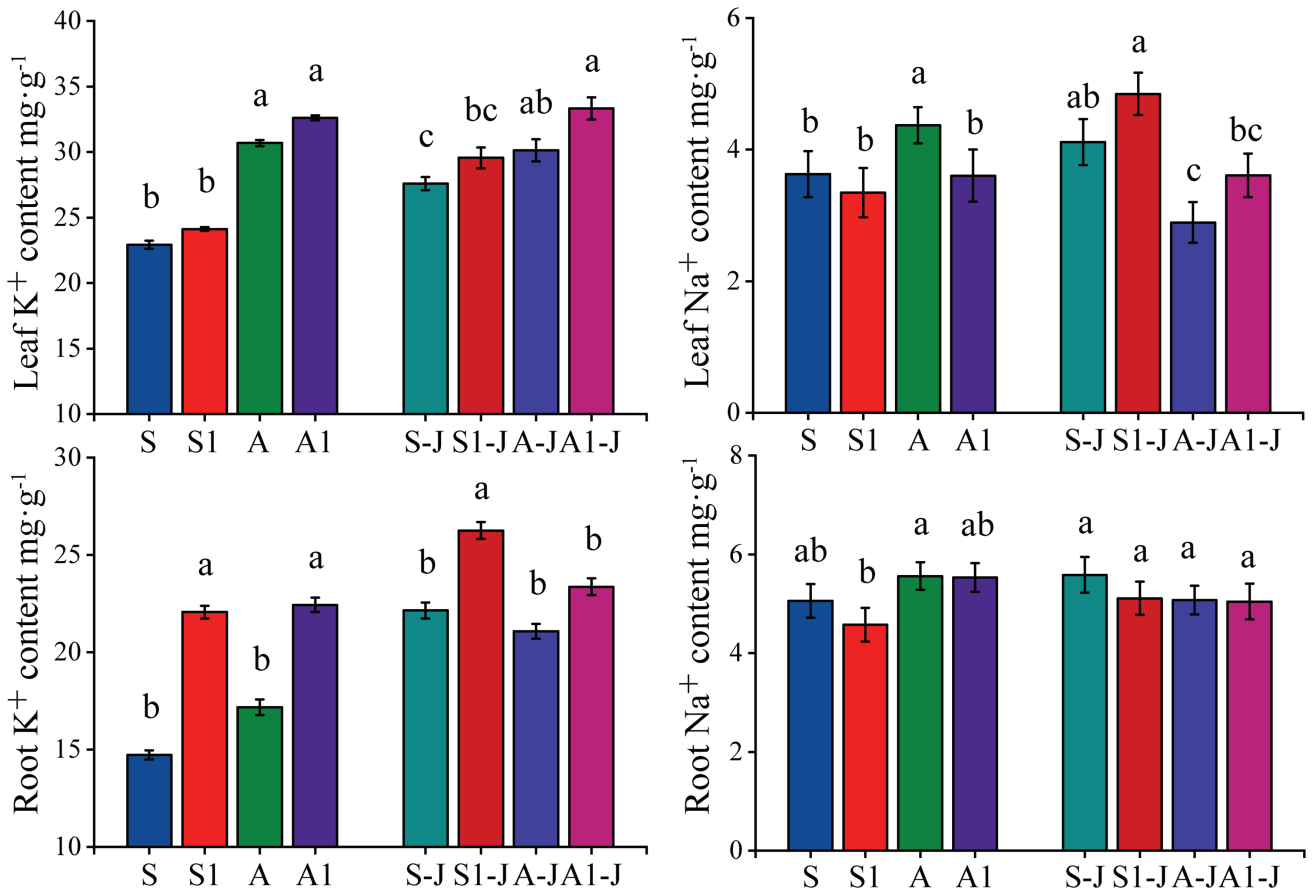
Effects of compound material on K^+^ and Na^+^ contents in leaf and root of cotton under saline and alkaline stresses. S, S1 indicate no compound material was applied in salinized soil, organic polymer was applied in salinized soil, respectively; A, A1 indicate no compound material was applied in alkalized soil, organic polymer was applied in alkalized soil, respectively. Bars represent SD of the mean (*n* = 3). Different lower-case letters indicate that there were significant differences among treatments at the level of 0.05.

For non-girdling cottons, the soluble sugar content in root for the A treatment was significantly increased by 38.04%, and no significant difference was found in the soluble sugar content in leaf, compared with those for the S treatment. The soluble sugar content in leaf for the S1 treatment was significantly increased by 62.14%, and no significant difference was found in the soluble sugar content in root, compared with those for the S treatment. The soluble sugar content in root for the A1 treatment was significantly increased by 49.87%, and no significant difference was found in the soluble sugar content in leaf, compared with those for the A treatment. For stem-girdling cottons, the soluble sugar content in leaf for the S1-J treatment was significantly increased by 30.16%, and no significant difference was found in the soluble sugar content in root, compared with those for the S-J treatment. The soluble sugar content in leaf and root for the A1-J treatment was significantly increased by 14.94 and 11.17%, respectively, compared with those for the A-J treatment (Figure 4).

For non-girdling cottons, there were no significant differences in the soluble protein content in leaf and root between the A treatment and the S treatment. The soluble protein content in leaf for the S1 treatment was significantly increased by 24.72% compared with that for the S treatment, while no significant difference was found between the A1 treatment and the A treatment. The soluble protein content in root for the A1 treatment was significantly increased by 23.23% compared with that for the A treatment, while no significant difference was found between the S1 treatment and the S treatment. For stem-girdling cottons, the soluble protein content in leaf for the S1-J treatment was significantly increased by 47.68% compared with that for the S-J treatment, while no significant difference was found between the A1-J treatment and the A-J treatment. There were no significant differences in the soluble protein content in root between the S1-J treatment and the S-J treatment and between the A1-J treatment and the A-J treatment (Figure 4).

### Effects of OPCM and Stem Girdling on K^+^ and Na^+^ Distribution of Cotton Under Saline and Alkaline Stresses

#### The Content of K^+^ and Na^+^ in Cotton Organs

After the application of OPCM, the content of K^+^ in root and leaf were increased compared with that for the control, while the content of Na^+^ in leaf and root was decreased; the K^+^ and Na^+^ contents in leaf were higher than those in root. For non-girdling cottons, the content of K^+^ and Na^+^ in leaf for the A treatment were significantly increased by 33.82 and 20.60%, respectively compared with those for the S treatment, while no significant difference was found in the content of K^+^ and Na^+^ in root. The content of K^+^ in root for the S1 treatment was significantly increased by 49.84% compared with that for the S treatment, while no significant differences were found in the content of Na^+^ in root and K^+^ and Na^+^ in leaf. The content of K^+^ in root for the A1 treatment was significantly increased by 30.63% compared with that for the A treatment, while the content of Na^+^ in leaf was significantly decreased by 6.49%; there was no significant difference in the content of K^+^ in leaf and Na^+^ in root. For stem-girdling cottons, the content of K^+^ in root for the S1-J treatment was significantly increased by 18.52% compared with that for the S-J treatment, while no significant differences were found in the content of K^+^ in leaf and Na^+^ in root and leaf. There was no significant differences in the content of K^+^ and Na^+^ in root and leaf between the A1-J treatment and the A-J treatment (Figure 2).

#### K^+^/Na^+^ Ratio for Cotton Organs

**Figure 5:**
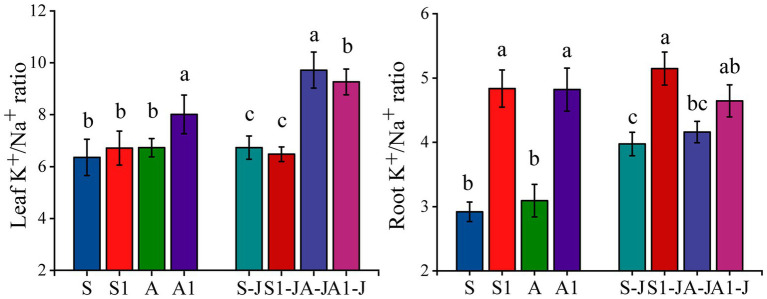
Effects of compound material on K^+^/Na^+^ ratio of leaf and root under saline and alkaline stresses. S, S1 indicate no compound material was applied in salinized soil, organic polymer was applied in salinized soil, respectively; A, A1 indicate no compound material was applied in alkalized soil, organic polymer was applied in alkalized soil, respectively. Bars represent SD of the mean (*n* = 3). Different lower-case letters indicate that there were significant differences among treatments at the level of 0.05.

For non-girdling cottons, there was no significant differences in the K^+^/Na^+^ ratio in leaf and root between the A treatment and the S treatment. The K^+^/Na^+^ ratio in root for the S1 treatment was significantly increased by 65.77% compared with that for the S treatment, while no significant difference was found in the K^+^/Na^+^ ratio in leaf. The K^+^/Na^+^ ratio in root and leaf for the A1 treatment were significantly increased by 55.84 and 19.10%, respectively compared with those for the A treatment. The K^+^/Na^+^ ratio of cotton organs changed significantly under saline and alkaline stresses. For stem-girdling cottons, the K^+^/Na^+^ ratio in root for the S1-J treatment was significantly increased by 29.51% compared with that for the S-J treatment, while no significant difference was found in the K^+^/Na^+^ ratio in leaf. The K^+^/Na^+^ ratio in leaf for the A1-J treatment was significantly increased by 4.68% compared with that for the A-J treatment, while no significant difference was found in the K^+^/Na^+^ ratio in root (Figure 5).

#### Selectivity Coefficients of K^+^ and Na^+^ Transport for Cotton Organs

For non-girdling cottons, there was no significant difference in the ST_K-Na_ of stem-root and leaf-stem between the A treatment and the S treatment. The ST_K-Na_ of leaf-stem for the S1 treatment was significantly increased by 39.59% compared with that for the S treatment, while the ST_K-Na_ of stem-root was significantly decreased by 54.47%. The ST_K-Na_ of stem-root for the A1 treatment was significantly decreased by 37.27% compared with that for the A treatment, and no significant difference was found in the ST_K-Na_ of leaf-stem. For stem-girdling cottons, the ST_K-Na_ of stem-root for the S1-J treatment was significantly decreased by 37.55% compared with that for the S-J treatment, while no significant difference was found in the ST_K-Na_ of leaf-stem. There was an opposite result in the alkalized soil. The ST_K-Na_ of leaf-stem for the A1-J treatment was significantly increased by 32.53%, while the ST_K-Na_ of stem-root was significantly decreased by 35.54%, compared with those for the A-J treatment (Table 3).

**Table 3:** Effects of compound material on K^+^ and Na^+^ transport coefficients of cotton organs under saline and alkaline stresses.

Treat	ST_K-Na_(leaf/stem)	ST_K-Na_(stem/root)
S	0.69 ± 0.09 b	3.17 ± 0.08 a
S1	0.96 ± 0.06 a	1.44 ± 0.04 c
A	0.65 ± 0.08 b	3.36 ± 0.05 a
A1	0.79 ± 0.05 ab	2.11 ± 0.10 b
S-J	0.46 ± 0.04 c	3.69 ± 0.09 a
S1-J	0.55 ± 0.06 c	2.31 ± 0.05 c
A-J	0.70 ± 0.01 b	3.36 ± 0.17 b
A1-J	0.92 ± 0.03 a	2.16 ± 0.06 c

S, S1 indicate no compound material was applied in salinized soil, organic polymer was applied in salinized soil, respectively; A, A1 indicate no compound material was applied in alkalized soil, organic polymer was applied in alkalized soil, respectively. Values are means ± SD of three independent replications (*n* = 3). Different lower-case letters indicate that there were significant differences among treatments at the level of 0.05.

### Redundancy Analysis

**Figure 6:**
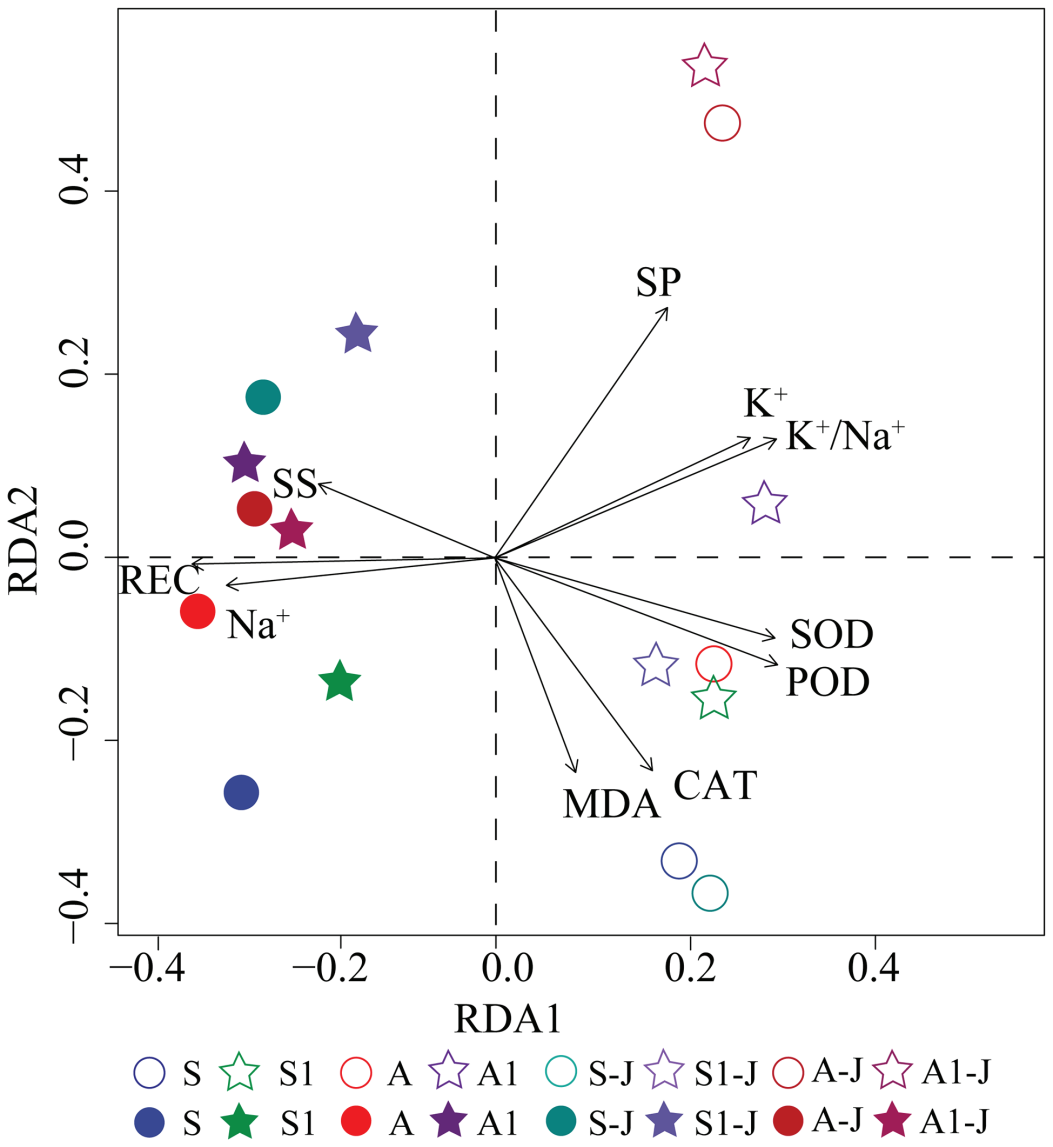
Redundancy analysis (RDA) of ion content and physiological characteristics of cotton under saline and alkaline stresses. Hollow legend represents leaf, and solid legend represents root. S, S1 indicate no compound material was applied in salinized soil, organic polymer was applied in salinized soil, respectively; A, A1 indicate no compound material was applied in alkalized soil, organic polymer was applied in alkalized soil, respectively.

Redundancy analysis was used to analyze the effects of OPCM on K^+^ and Na^+^ absorption and physiological characteristics of leaf and root of cottons under saline and alkaline stresses. The results showed that root and leaf was separated by the RDA1 axis. CAT, MDA, and soluble protein (SP) had the highest correlations with the RDA1 axis, and SOD, POD, soluble sugar (SS), K^+^ content, Na^+^ content, K^+^/Na^+^ ratio, and relative electrical conductivity (REC) had the highest correlations with the RDA2 axis (Figure 6). The SOD, POD, CAT, MDA, K^+^ content, SP, and K^+^/Na^+^ ratio were obviously distributed on the right side of the RDA1 axis; the soluble sugar, Na^+^ content, and REC were obviously distributed on the right side of the RDA2 axis. Na^+^, REC, and SS were closely related to the root for all treatments, and SOD, POD, CAT, MDA, SP, K^+^, and K^+^/Na^+^ ratio were closely related to the leaf for all treatments.

## Discussion

Recent studies have shown that exogenous application of betaine and vanillic acid could significantly improve the salt tolerance of crops (Parvin et al., 2020), and increase the yields (Sofy et al., 2020). In this study, for non-girdling cottons, no matter under saline stress or alkaline stress, the application of OPCM promoted the growth of cotton in different degrees and significantly increased the boll weight, which was consistent with the study results of Alam et al. (2019) and Ahanger et al. (2019) have shown that exogenous application of kinetin (a cytokinin) could increase the activities of enzymes, ascorbic acid, and glutathione under salt stress. For the cotton without girlding in this study, it was also found that the application of OPCM significantly reduced the content of MDA in leaf, and the activities of SOD, POD, and CAT in leaf under saline stress and the activities of SOD and POD in leaf under alkaline stress were significantly increased, which were consistent with the study results of Kaya et al. (2020; Figure 3). Moreover, the content of soluble sugar and soluble protein in cotton leaf and root were all increased under saline and alkaline stresses (Figure 4). This indicated that the effect of the OPCM on enzyme activities were the similar under saline and alkaline stresses. The increases of SOD, POD, and CAT activities and the soluble sugar and soluble protein contents could reduce or eliminate the damage of reactive oxygen species on the membrane, and reduce the accumulation of MDA, thus protecting the membrane structure (Ren et al., 2020). The saline and alkaline stresses could be alleviated through regulating antioxidant enzymes to improve plant antioxidant capacity (Ahmad et al., 2010). However, in this study, the effect of OPCM on the growth of cotton under saline stress was greater than that on the growth of cotton under alkaline stress, which was consistent with the results of Dong et al. (2014). For stem-girdling cottons, the activities of SOD and CAT and the content of REC, soluble protein, and soluble sugar in cotton leaf and root were also increased under saline and alkaline stresses, while the content of MDA in leaf was decreased. The effect of OPCM on cotton leaf and root under alkaline stress was greater than that under saline stress. Mora et al. (2009) have reported that the application of manganese could increase the antioxidant enzyme activity and lower the lipid peroxidation of ryegrass. Zhang et al. (2013) have found that SOD activity is positively correlated with Zn^2+^ content. Chen et al. (2005) have found that the Mn^2+^ and Zn^2+^ in the OPCM could be released slowly to effectively alleviate the saline stress and alkaline stress on the antioxidant enzyme activities and osmotic adjustment substances of cotton.

Under saline and alkaline stresses, the OPCM not only improved the physiological and biochemical indexes of cotton organs, but also regulated the transport efficiency of K^+^ and Na^+^. The difference in physiological damage could also affect the K^+^ and Na^+^ content of cotton under saline and alkaline stresses (Elgendy et al., 2015). Many studies have shown that exogenous application of Si and glutathione (GSH) can significantly regulate the content of K^+^ and Na^+^ in plants (Ahmad et al., 2018) and the ion imbalance caused by saline and alkaline stresses, and improve the salt tolerance of plants (Parvin et al., 2020). Through the experiments of stem-girdling, it was found that the absorption of K^+^ and Na^+^ in different organs of cotton were different under saline and alkaline stresses, leading to the significant difference in the transport efficiency of K^+^ and Na^+^. The application of OPCM increased the K^+^/Na^+^ ratio in leaf and root and decreased the content of Na^+^. This indicated that the OPCM could regulate the absorption and distribution of K^+^ and Na^+^ in cotton, inhibits the absorption of Na^+^ by stem, and enhances the transport of K^+^ from stem to leaf, to maintain the K^+^ and Na^+^ homeostasis in different organs of cotton under saline and alkaline stresses. However, the transport efficiency of K^+^ and Na^+^ from stem to leaf under saline stress was higher than that under alkaline stress. It may be due to that alkaline stress not only causes ion toxicity and osmotic stress, but also causes high pH, compared with saline stress, which results in the greater negative effect on the transport efficiency (Yang et al., 2008). Therefore, the effect of OPCM on cotton under saline stress is more significant than that on cotton under alkaline stress. The RDA analysis further showed that the activities of Na^+^, K^+^/Na^+^ ratio, REC, SOD, and soluble sugar content were significantly affected by the OPCM under saline and alkaline stresses. The regulating effect of OPCM on leaf and root were different. OPCM mainly affected the activities of SOD, POD, and K^+^/Na^+^ ratio in leaf, and the content of Na^+^, REC, and soluble sugar in root. For stem-girdling cottons, the content of K^+^ and Na^+^ in leaf were increased, and the content of Na^+^ in root was decreased; moreover, the regulation effect of stem girdling on ion homeostasis in leaf and root was more prominent under saline stress, indicating that the regulation mechanism of ion homeostasis by stem girdling are different under saline stress and alkaline stress (Ma et al., 2020). Although stem girdling reduced the K^+^ and Na^+^ transport efficiency, the application of OPCM maintained the ion homeostasis in organs (An et al., 2019). Through RDA analysis, it was found that the effect of OPCM on the activities of SOD and POD in leaf and REC, Na^+^, and soluble sugar contents in root of cottons with stem girdling were consistent with that on those of cottons without stem girdling.

To further study the dynamic change of K^+^ and Na^+^, the ion selective coefficient (ST_K-Na_) were calculated. It was found that although the transport efficiency of K^+^ and Na^+^ in organs of cotton with stem girdling was lower than that in organs of cotton without stem girdling, there was still a promoting effect (Table 3). This may be due to the fact that although the phloem of main stem was cut off by girdling, K^+^ and Na^+^ in root could be transported to the aboveground part through the xylem due to the root pressure and transpiration, leading to a new ion homeostasis in leaf and root (Singh et al., 2015). The influences on ion transport in cotton leaf and root by OPCM is due to the three-dimensional network structure of polymer materials in the OPCM. The hydrophilic functional groups, which can absorb a large amount of water and saline solution, inhibit the loss of water through evaporation in salinized and alkalinized soils (Ni et al., 2010). At the same time, saline and alkaline stresses cause the dissociation of carboxylic acid groups in polymer materials. Due to the shielding effect of Na^+^ on carboxylic acid anions, the anion exclusion between carboxylic acid groups could be hindered (Gharekhani et al., 2017), which could effectively alleviate the ion damage caused by saline and alkaline stresses, and maintain the ion homeostasis in cotton organs.

## Conclusion

In this study, the analysis of cotton without girdling treatment showed that the OPCM had different effects on saline stress and alkaline stress on cotton. The Na^+^ content in leaf and root of cotton under saline stress were lower than those of cotton under alkaline stress. The OPCM could improve the activities of POD and CAT, and further regulate the transport efficiency of K^+^ and Na^+^ in cotton leaf and root, to reduce the saline and alkaline stresses on cotton. The analysis of cotton with girdling treatment showed that the OPCM could increase the activities of SOD and CAT in leaf and root and the K^+^ content, and improve the saline and alkaline tolerance of cotton. The results further confirmed the potential of our self-developed OPCM in regulating NaCl stress and Na_2_CO_3_ stress on cotton.

## Data Availability Statement

The original contributions presented in the study are included in the article/Supplementary Material, further inquiries can be directed to the corresponding author.

## Author Contributions

KW, XW, and MA conceived, designed, and conducted the experiments. JS and KC helped in conducting experiments. XW analyzed the data results and wrote the manuscript. KW and HF monitored the experimental work and critically commented on the manuscript. All authors contributed to the article and approved the submitted version.

### Conflict of Interest

The authors declare that the research was conducted in the absence of any commercial or financial relationships that could be construed as a potential conflict of interest.
